# The utility of salivary CRP and IL-6 as a non-invasive measurement evaluated in patients with COVID-19 with and without diabetes

**DOI:** 10.12688/f1000research.130995.2

**Published:** 2023-10-25

**Authors:** Endang Bachtiar, Boy M Bachtiar, Ardiana Kusumaningrum, Hari Sunarto, Yuniarti Soeroso, Benso Sulijaya, Efa Apriyanti, Citra Fragrantia Theodorea, Irandi Putra Pratomo, Yudhistira Yudhistira, Defi Efendi, Widya Lestari

**Affiliations:** 1 Department of Oral Biology and Oral Sciences Research Center, Faculty of Dentistry Universitas Indonesia, Jakarta, Indonesia, 10430, Indonesia; 2 Department of Microbiology, Faculty of Medicine, Universitas Indonesia; Clinical Microbiology Medicine Staff Group, Universitas Indonesia Hospital, Jakarta, Indonesia, 10430, Indonesia; 3 Department of Periodontology, Faculty of Dentistry, Universitas Indonesia, Jakarta, Indonesia, 10430, Indonesia; 4 Dental Center, Universitas Indonesia Hospital, Depok, West Java, Indonesia; 5 Department of Pediatric Nursing, Faculty of Nursing Universitas Indonesia, and Paediatric Intensive Care Unit, Universitas Indonesia Hospital, West Java, Indonesia; 6 Department of Pulmonology and Respiratory Medicine, Faculty of Medicine, Universitas Indonesia, Salemba Raya 6, Jakarta, 10430, Indonesia; 7 Clinical Pathology Medicine Staff Group,, Universitas Indonesia Hospital., Depok, West Java, Indonesia; 8 Department of Pediatric Nursing, Faculty of Nursing Universitas Indonesia, and Neonatal Intensive Care Unit, Universitas Indonesia Hospital, Depok, West Java, Indonesia; 9 Oral Biology Unit, Fundamental Dental and Medical Sciences Kuala Lumpur, Malaysia International Islamic University Malaysia, Kuala Lumpur, Malaysia

**Keywords:** Diabetes, COVID-19, periodontitis, C-reactive protein, interleukin-6

## Abstract

**Background:** The available evidence suggests that inflammatory responses, in both systemic and oral tissue, contribute to the pathology of COVID-19 disease. Hence, studies of inflammation biomarkers in oral fluids, such as saliva, might be useful to better specify COVID-19 features.

**Methods**: In the current study, we performed quantitative real-time PCR to measure salivary levels of C-reactive protein (CRP) and interleukin-6 (IL-6) in saliva obtained from patients diagnosed with mild COVID-19, in a diabetic group (DG; n = 10) and a non-diabetic group (NDG; n = 13). All participants were diagnosed with periodontitis, while six participants with periodontitis but not diagnosed with COVID-19 were included as controls.

**Results:** We found increases in salivary total protein levels in both the DG and NDG compared to control patients. In both groups, salivary CRP and IL-6 levels were comparable. Additionally, the levels of salivary CRP were significantly correlated with total proteins, in which a strong and moderate positive correlation was found between DG and NDG, respectively. A linear positive correlation was also noted in the relationship between salivary IL-6 level and total proteins, but the correlation was not significant. Interestingly, the association between salivary CRP and IL-6 levels was positive. However, a moderately significant correlation was only found in COVID-19 patients with diabetes, through which the association was validated by a receiver operating curve.

**Conclusions:** These finding suggest that salivary CRP and IL-6 are particularly relevant as potential non-invasive biomarker for predicting diabetes risk in mild cases of COVID-19 accompanied with periodontitis.

## Introduction

There is a growing interest in measuring inflammatory biomarkers in saliva, as sampling this oral fluid is less invasive than blood. ^1^ ^-^ ^3^ Despite the promising results of saliva studies, the findings of salivary measures of inflammation have been inconsistent.

In this study, we used saliva derived from patients with COVID-19 to evaluate the association of selected inflammatory markers, interleukin-6 (IL-6), and C-reactive protein (CRP) in COVID-19 patients. The extent to which salivary IL-6 and CRP levels are associated with diabetes or non-diabetes events in this population still needs to be explored.

## Methods

### Subjects, saliva sampling, and in vitro methods

This study was part of a project on COVID-19 and its association with the oral ecosystem. Thus, the current report was based on data from our previous study, in which data from 23 people with mild severe acute respiratory syndrome coronavirus 2 (SARS-CoV-2) infection were requested from Rumah Sakit Universitas Indonesia (RSUI), a university hospital, between June and August 2021. ^4^ In addition to COVID-19, all patients were diagnosed with periodontitis. Patients were further divided into two groups: with (n = 10) and without (n = 13) diabetes. Six non-diabetic participants without COVID-19 and no periodontitis were included as controls. Sampling methods and information regarding the inclusion and exclusion criteria of patients, how consent was obtained from participants, and obtaining medical reports were performed in accordance with the guidelines provided by the ethics committee of RSUI (protocol number: 2021/04/052). All of the participants signed the written informed consent form. Additionally, this study was performed in accordance with the Strengthening the Reporting of Observational Studies in Epidemiology (STROBE) guidleines. ^5^ Information regarding clinical status, such as age, sex, and chronic medical history of comorbidities, was obtained from the medical reports of mildly symptomatic patients with COVID-19 (not shown). Only subjects who had no respiratory symptom for more than 2 weeks were included in this study. However, our focus was on patients with COVID-19, with and without diabetes. Thus, only diabetic status (type 2 diabetes), as reported in the medical records, was included as a comorbidity variable in the data analysis, while all COVID-19 patients were diagnosed with periodontitis (moderate to severe) according to the criteria described by the American Academic of Periodontology Classification of Periodontal Disease, ^6^ without dental radiographic assessment.

The oral specimen was obtained by spitting unstimulated whole saliva into a sterile Falcon tube, placed on ice, and transferred to the laboratory for subsequent processing. ^4^

The total protein level in saliva was determined using the Bradford assay method, as reported elsewhere. ^7^ The concentration of CRP in saliva samples was determined using an enzyme-linked immunosorbent assay kit (Elabscience Biotechnology Inc., Wuhan, China) according to the manufacturer’s instructions. For IL-6, we used quantitative real time-polymerase chain reaction (qPCR; ABI StepOnePlus Real-Time PCR system), using SYBR Green I for gene expression analysis, to amplify cDNA that had been converted from salivary RNA using the method we used for the gingival crevicular sample. ^8^ The PCR was run in triplicate using primers (IL-6 and housekeeping gene/GAPDH) and the PCR program as reported previously. ^8^ The 2 ^-ΔΔCT^ method was used to analyze the relative expression of mRNA. ^9^

### Data analysis

There was a strong correlation between mRNA and protein expression levels. ^10^ In this study, owing to the observed variations in saliva concentrations, both CRP (protein) and IL-6 (transcription level) were adjusted for total salivary protein. Statistical analyses were performed using GraphPad Prism 9.4 software. Data are presented as mean ± SD or median. The nonparametric Kruskal Wallis test was used to compare the diabetic group (DG), the non-diabetic group (NDG), and the control group. The Mann-Whitney test was used to compare the DG and NDG data. The statistical significance level was set at p < 0.05. Spearman’s correlation coefficient (r) with two-tailed p-values was calculated, and linear regression was used to generate the line of best fit with 95% confidence intervals. Receiver operating characteristic (ROC) curve analysis was also performed to evaluate this association.

## Results

**Figure 1.**
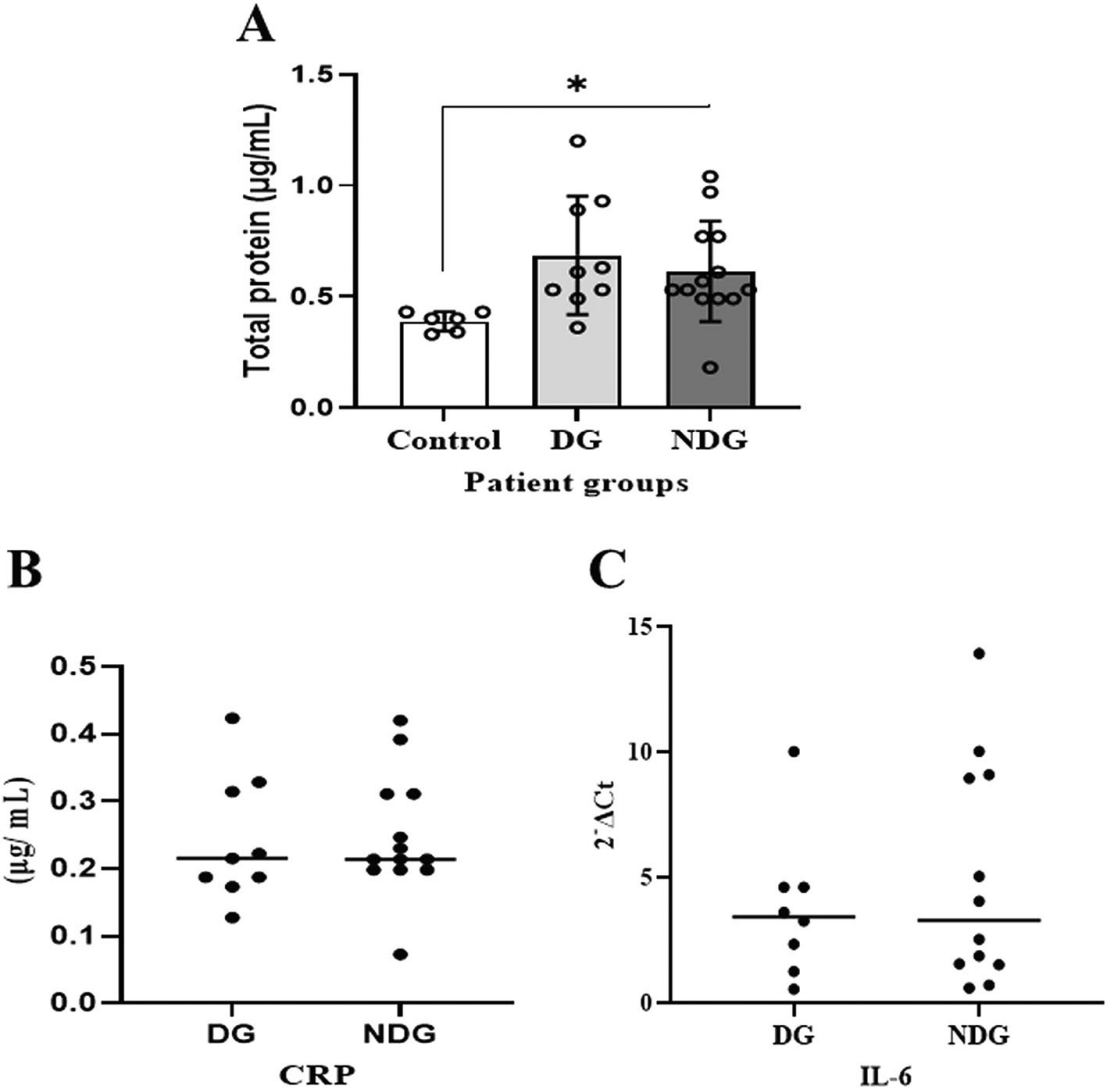
Comparison of total salivary protein concentration in patients with COVID-19 with and without diabetes. Significant differences in salivary total proteins concentration were observed in both DG and NDG groups compared to control (A). However, in each group tested, the salivary level of either CRP (B) or IL-6 (C) was comparable, as assessed by Bredford protein assay and quantification of mRNA expression, respectively. *p < 0.05. CRP = C-reactive protein, Il-6 = Interleukin-6. DG and NDG are diabetes and non-diabetes group, respectively.

As shown in Figure 1A, compared to the control group, the mean values of the total protein concentration in the saliva of the DG and the NDG were significantly increased (p < 0.05). However, the concentrations were comparable in both groups.

We further noted that in the DG, salivary CRP was detectable in 9/10 (90%) patients, while IL-6 was only measured in 8/10 (80%) patients. In the NDG, the concentration of CRP and the transcription level of IL-6 in saliva samples were detected in all (100%) and 12/13 (92.31%), respectively. The median values of CRP and IL-6 in saliva are shown in Figure 1B, C. The protein concentrations of CRP and the transcription level of IL-6 mRNA detected in saliva among the patient groups were comparable (p > 0.05).

**Figure 2.**
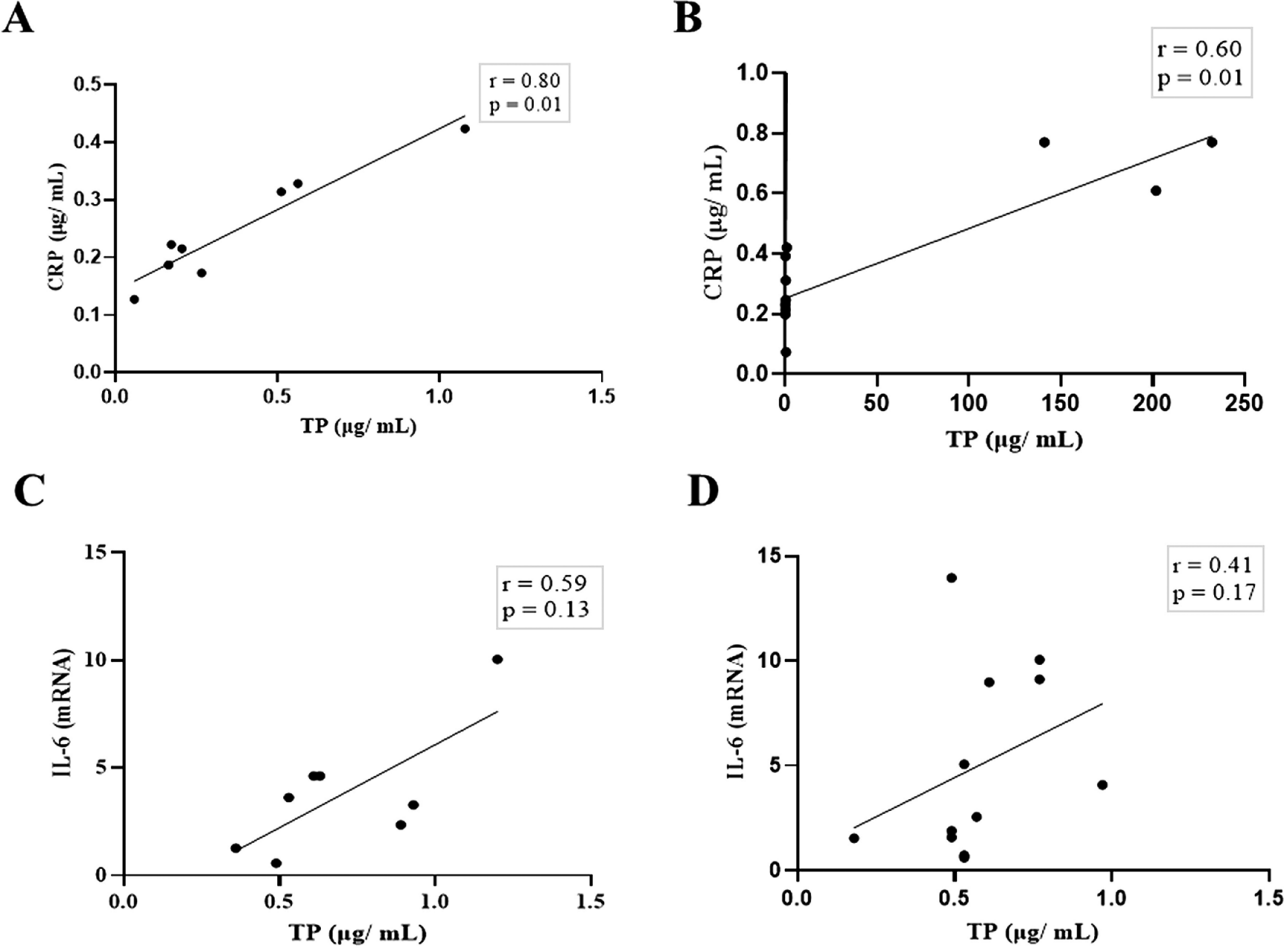
Relationship between salivary levels of CRP/IL-6 and total protein in patients with COVID-19 with and without diabetes. Spearman correlation analysis indicates a statistically significant positive correlation between salivary levels of CRP and total protein, A strong and moderate correlations was observed in DG (A) and NDG (B), respectively. The positive relationship between IL-6 salivary levels and total salivary proteins were also found in DG (C) and NDG (D). However, the correlations were not significant. CRP = C-reactive protein, IL-6 = Interleukin-6. DG and NDG. Spearman correlation coefficient (r) and exact p-value are shown.

**Figure 3.**
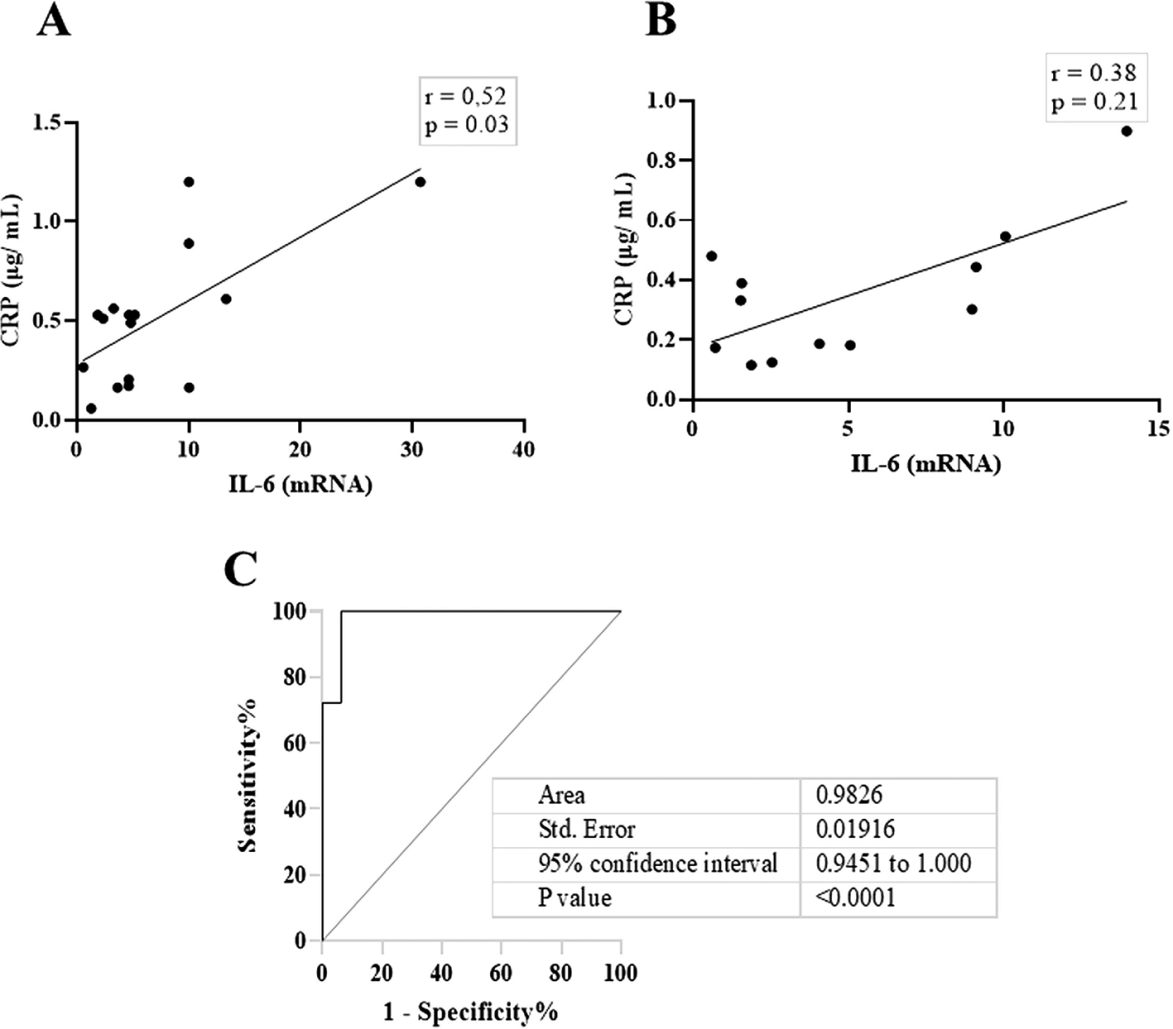
The scatter diagram denotes the relationship between salivary levels of CRP and IL-6. These data indicate that in DG (A), the correlation was significantly moderate, while a weak not significant correlation was noted in NDG (B). Receiver operating characteristic (ROC) was used to illustrate the plot and best cut-off of significant relationship between salivary levels of CRP and Il-6 in DG group (C). CRP = C-reactive protein, IL-6 = Interleukin-6. DG and NDG. Spearman correlation coefficient (r) and exact p-value are displayed.

Next, we compared the association between total salivary proteins and each of the two inflammation markers detected in the saliva. As shown in Figure 2A–D, IL-6 and CRP levels were identical in their association with total salivary proteins. Spearman’s correlation showed a strongly significant positive correlation between salivary CRP and total salivary proteins in the DG (r = 0.80, p = 0.01), while a moderately significant correlation was observed in the NDG (r = 0.60, p = 0.01). A similar trend was observed for the correlation between salivary IL-6 and total salivary protein levels. In the DG (r = 0.59, p = 0.13) and the NDG (r = 0.41, p = 0.17), the correlation was positive, but not significant. Finally, we compared the correlation between the two inflammation markers in each group. In both groups, the correlation was positive, but a significant association was only observed in the DG. The correlation coefficients were r = 0.52, p = 0.03, and r = 0.38, p = 0.21 in the DG and NDG, respectively ( Figure 3A,B). Hence, since a significant relationship was only shown in the DG, we made inferences about association accuracy by performing ROC analysis. The area under the curve of the CRP/IL-6 association was 0.983 (95% confidence interval [CI]: 0.9451–1, p < 0.0001; Figure 3C).

## Discussion

The most obvious finding of this study is that salivary CRP and IL-6 are potential markers of systemic inflammation in mild cases of COVID-19 with and without diabetes. With regard to CRP, our results were not consistent with a recent report, which showed that an increased concentration of salivary CRP was only found in severe cases of COVID-19. ^11^ We reasoned that this inconsistency could be related to the eligibility criteria in recruiting participants. In the cited report, the authors excluded participants with periodontitis, whereas in our study, periodontitis was an inclusion criterion. ^8^ However, for IL-6, the results of this study are in line with a previous report, where the concentration of salivary IL-6 was elevated in periodontitis patients with and without diabetes. ^12^

Current literatures show that IL-6 is a pleiotropic cytokine with complex role in inflammation and metabolic disease. Its biological activities include B-lymphocyte differentiation, T-lymphocyte proliferation, ^13^ pro and anti-inflammatory activities, ^14^ the development of the nervous and hematopoietic system, and the regulation of metabolism. ^15^ As a proinflammatory cytokine, it induces insulin resistance and periodontal disease in the process of bone resorption. ^16^ Taken together, Il-6, which is considered an adipokine has an important role in the pathogenesis of localized oral inflammation (periodontitis), and may play a pivotal role in metabolic disease, ^17^ such as diabetes.

The current study is an extension of our primary study involving the same patients with COVID-19 to evaluate the inflammatory conditions in periodontal microenvironment. ^18^ We found that the transcription levels of both IL-6 and complement C3 (the central component of innate immune system) in gingival crevicular fluid (GCF) were markedly higher in COVID-19 patients with diabetes (DG) compared to the non-diabetes patients (NDG). We also noticed that in periodontal niche, the mRNA upregulation of host receptor for SARS-CoV-2 (angiotensin-converting enzyme 2/ACE2) ^19^ was positively associated with the transcription levels of either inflammation marker tested (IL-6 or C3). Thus, our previous results along with other work by other investigators ^20^ ^,^ ^21^ suggest that the virus receptor (ACE2) can be detected in oral tissue. Additionally, certain molecules (furin and TMPRSS2) that are involved in promoting the SARS-CoV-2 entry and infection can be detected in oral cavity. ^22^ This means that the essential molecules for SARCOV-2 infection are abundant in the oral cavity, and the infected virus may lead to localized inflammation and loss of taste (dysgeusia) as well as dry mouth, which are the most frequently reported symptom in COVID-19 patient. ^23^ ^–^ ^25^ The literature search also indicated that the relationship between COVID-19 and diabetes mellitus is complicated and bidirectional. ^26^ In addition to diabetes, all patients with COVID-19 included in the current study also had periodontitis, a common comorbidity observed in patients with COVID-19. ^27^

Consequently, it is necessary to consider the influence of confounding variables on the oral ecology. Therefore, to optimize the utility of salivary CRP and Il-6 as inflammation markers in separating patients with COVID-19 with and without diabetes, we used total salivary protein concentration to normalize the tested analyte levels (CRP and IL-6) in saliva. ^28^

Compared to the control group (non-SARS-CoV-2 infected participants), we observed a highly significant increase in the salivary total protein concentration in both patient groups tested. These results suggest that elevated total salivary protein concentration occurred in all patients with COVID-19. Therefore, we assumed that the rising total salivary protein levels in our patients with COVID-19 might include increasing levels of both salivary inflammation markers (CRP and IL-6).

Based on the design mentioned above, we noticed the association between salivary CRP/IL-6 and total salivary proteins was consistent, but the strength of the correlation varied. In both patient groups, a significantly positive association was found only for CRP. A strong and moderate correlation was observed in the DG and the NDG. Further, moderate, and weak positive correlations between IL-6 and total protein were found in the DG and the NDG, respectively. Overall, these results indicates that the highest correlation (0.80) was found between salivary CRP and total protein of hospitalized COVID-19 patients with diabetes, while the non-diabetic patients had a lower correlation of 0.60. Since CRP in oral fluids reflects its level in circulation, ^29^ our result might indicate that in COVID-19 patients with diabetes, the proportion of salivary CRP was greater when serum levels were raised.

However, unlike salivary CRP, IL-6 levels in saliva have been reported to not correlate with those in plasma or serum. ^30^ ^,^ ^31^ In this regard, the elevated levels observed in this study may indicate inflammation induced by periodontitis-associated bacteria, particularly *Porphyromonas gingivalis*, as its components (dipeptidyl peptidase-4/DPP4), that mimic human dpp4, involved in postprandial glycemic control in individuals with type 2 diabetes, ^32^ ^,^ ^33^ that according to the medical record, were the participants included in the current study.

Moreover, it seems likely that IL-6 is a key stimulator of the hepatic synthesis of CRP. ^34^ In contrast, the salivary levels of CRP and IL-6 appeared to be independently regulated by diabetes status. As indicated in this study, a positive correlation between the two cytokines was observed in patients with and without diabetes.

This result was also corroborated by the ROC curve, and we were able to attain nearly 100% sensitivity and specificity. We believe that defining the relationship between CRP and IL-6 in patients with COVID-19 is unique to separate diabetes and non-diabetes in patients with COVID-19 suffering from periodontitis as a comorbidity.

Taken together, as mentioned above, the salivary CRP level reflects the CRP level in circulation, and it is likely that our data may reflect a positive correlation between IL-6 and CRP levels in the serum. Therefore, the results of the current study suggest that salivary levels of IL-6 might be overshadowed by local inflammation (periodontitis) instead of inflammation-related diabetes. Indeed, our findings provide additional information regarding the unequivocal results with either the presence ^35^ ^,^ ^36^ or the absence ^37^ of a relationship between the status of periodontal disease and salivary CRP.

Considering that CRP is a key cytokine that plays an important role in the progression of various inflammatory diseases, ^38^ we assumed that both periodontitis and diabetes are related to the pathophysiology of COVID-19. This association could be linked to the existence of two unique protein structures in CRP. ^39^ The first isoform is a CRP monomer that is activated by local signals of inflammation and tissue injury, and the other is the pentameric isoform synthesized by the liver. ^40^ ^,^ ^41^ As our participants were patients with COVID-19 with and without diabetes accompanied by periodontitis, we assumed that the salivary CRP detected in this study is the monomeric isoform that might have been activated by periodontal inflammation. Its presence in saliva may have been independently regulated by the diabetes status of our patients. Further studies are required to clarify this assumption.

Alternative explanations for our results warrant further discussion. First, SARS-CoV-2 infection is closely related to depressive disorder, ^42^ ^,^ ^43^ and the positive correlation between salivary CRP and IL-6 levels observed in this study may indicate a characteristic inflammatory response in depressive disorder that is commonly found in COVID-19 patients. ^44^ Second, since diabetes is among the common comorbidities noted in patients diagnosed with COVID-19, ^45^ the similar effect of the depression-associated inflammatory response (salivary CRP and IL-6) found in the two tested groups (DG and NDG) might be a consequence of the impact of depression on SARS-CoV-2 infection rather than diabetes itself. Thus, it is possible that the subtle elevation of the selected salivary biomarkers observed in the DG and the NDG (shown in Figure 2B, C) was no longer statistically significant. Further studies are required to confirm this possibility.

This observational study has some limitations. First, the included participants were restricted; therefore, further studies including more participants are required. Second, the changes in salivary proteins during the time saliva was collected was not part of this study design, so future studies are necessary to assess dynamic salivary changes. Lastly, we cannot rule out the involvement of salivary flow rate in determining salivary CRP and IL-6 levels. Therefore, it is recommended to include this confounding variable as a patient-specific factor in future investigations and to confirm the results of this study.

## Conclusions

The current study has shown that the low correlation between salivary levels of CRP and IL-6 seems to be a valuable oral fluid biomarker to discriminate between diabetic and non-diabetic status in patients with COVID-19. Additionally, the finding in saliva analytes (CRP and IL-6) coincides with oral dysbiosis, which is represented by periodontitis status. ^4^ This suggests that in cases of mild SARS-CoV-2 infection, oral dysbiosis in individuals with diabetes might increase the probability of diabetes-associated inflammation, which is characterized by increased levels of salivary CRP and IL-6. Prospective studies are needed to determine the feasibility of salivary CRP/IL-6 as a prognostic measure for the risk of systemic and oral inflammation.

## Data availability

### Underlying data

Open Science Framework: The utility of salivary CRP and IL-6 as a non-invasive measurement evaluated in patients with COVID-19 with and without diabetes, https://doi.org/10.17605/OSF.IO/PXD6R. ^46^

This project contains the followign underlying data:

• Excel data TP-CRP-IL-6 F1000 Jan 23.xlsx

Data are available under the terms of the Creative Commons Zero “No rights reserved” data waiver (CC0 1.0 Public domain dedication).
