# Supplementary material for: The utility of salivary CRP and IL-6 as a non-invasive measurement evaluated in patients with COVID-19 with and without diabetes
Source: F1000Res. 2024 Jan 11;12:419. Originally published 2023 Apr 18. [Version 3] doi: 10.12688/f1000research.130995.3 (PMC10806364; doi:10.12688/f1000research.130995.3)
Supplement: Peer review history

# Reviewer report: Reviewer response for version 3

**DOI:** 10.5256/f1000research.161123.r237285

**Published:** 2024-01-23

**Authors:** Ghada Ibrahim Taha

**Affiliations:** 1 University of Baghdad, Baghdad, Baghdad Governorate, Iraq

Revised accepted

Is the work clearly and accurately presented and does it cite the current literature?

No

If applicable, is the statistical analysis and its interpretation appropriate?

Yes

Are all the source data underlying the results available to ensure full reproducibility?

Yes

Is the study design appropriate and is the work technically sound?

Partly

Are the conclusions drawn adequately supported by the results?

Partly

Are sufficient details of methods and analysis provided to allow replication by others?

Partly

Reviewer Expertise:

microbiology and Clinical immunologist

I confirm that I have read this submission and believe that I have an appropriate level of expertise to confirm that it is of an acceptable scientific standard.

# Reviewer report: Reviewer response for version 2

**DOI:** 10.5256/f1000research.157516.r232990

**Published:** 2024-01-04

**Authors:** Ghada Ibrahim Taha

**Affiliations:** 1 University of Baghdad, Baghdad, Baghdad Governorate, Iraq

Dear Author

 I have carefully reviewed your manuscript and would like to bring the following points to your attention for clarification and enhancement:

 Sample Clarification: The manuscript initially refers to the use of saliva samples; however, later sections mention a 'Gingival crevicular fluid (GCF)' in the context of interleukin-6 detection. Could you please clarify the exact nature of the samples used? If the GCF is indeed the sample of choice, kindly detail its collection method and the protocol employed in your study.

 Sample Size Justification: The number of samples utilized in the study appears quite limited. Could you provide a rationale for this sample size? An explanation of how it's statistically sufficient to support the study's conclusions would be beneficial.

 Study Group Composition: The research seems to encompass three distinct groups: individuals with gum disease, those with diabetes, and those affected by Covid. This approach appears somewhat broad. Could you elucidate whether the aim is to establish a correlation among these conditions? If so, references to prior studies establishing such a relationship would significantly strengthen your argument.

 Control Group Discrepancy: There seems to be a contradiction regarding the control group's condition; the summary indicates they suffer from gum disease, whereas the methods section suggests they are free from any ailments. A consistent definition of the control group across all sections is crucial for the clarity and integrity of the study.

 Supporting Evidence for Conclusions: The current conclusions would benefit greatly from additional supporting evidence from relevant studies. Incorporating references and discussions around existing research would provide a more robust context for your findings.

 Exploration of Limitations: I noticed an absence of a detailed discussion on the limitations of your study. Understanding and acknowledging these limitations is vital for a comprehensive view of the research's scope and impact.

 Your attention to these matters would greatly enhance the clarity and depth of your study. I look forward to your revisions.

 Best regards,

Is the work clearly and accurately presented and does it cite the current literature?

No

If applicable, is the statistical analysis and its interpretation appropriate?

Yes

Are all the source data underlying the results available to ensure full reproducibility?

Yes

Is the study design appropriate and is the work technically sound?

Partly

Are the conclusions drawn adequately supported by the results?

Partly

Are sufficient details of methods and analysis provided to allow replication by others?

Partly

Reviewer Expertise:

microbiology and Clinical immunologist

I confirm that I have read this submission and believe that I have an appropriate level of expertise to confirm that it is of an acceptable scientific standard, however I have significant reservations, as outlined above.

# Response

**Published:** 2024-01-07

**Authors:** Endang Bachtiar

Dear Reviewer,

 We appreciate the time and effort that you dedicated to providing feedback on our manuscript in this journal. We are grateful for the insightful comments on valuable improvements to our paper.

 We have incorporated most of your suggestions. Please see below, the point-by-point response to the reviewer’s comments and concerns

**Reviewers’ comments (RC) and Author Response (AR)**

  

1. **RC**: I have carefully reviewed your manuscript and would like to bring the following points to your attention for clarification and enhancement:

**AR**: Thank you.

        Sample Clarification: The manuscript initially refers to the use of saliva samples; however, later sections mention a 'Gingival crevicular fluid (GCF)' in the context of interleukin-6 detection. Could you please clarify the exact nature of the samples used? If the GCF is indeed the sample of choice, kindly detail its collection method and the protocol employed in your study.

**AR**: We appreciate the feedback provided by the reviewer. We therefore need to be clear that, as we explained in the Methods section, we amplified cDNA that had been converted from salivary RNA using the same procedure we used for the gingival crevicular sample for IL-6 using quantitative real-time polymerase chain reaction (qPCR; ABI StepOnePlus Real-Time PCR system) and SYBR Green I for gene expression analysis. ^8^ Thus, in this study we used saliva instead of GCF as oral sample.

  

1. **RC**: Sample Size Justification: The number of samples utilized in the study appears quite limited. Could you provide a rationale for this sample size? An explanation of how it's statistically sufficient to support the study's conclusions would be beneficial.

**AR**: The number of patients included in the study was limited  as we adhered to the committee's ethical guidelines that only patients with mild COVID-19 infection were allowed to have oral samples taken. We analyzed the collected data using a non-parametric test, as we indicated in the Methods section.

  

1. **RC**: Study Group Composition: The research seems to encompass three distinct groups: individuals with gum disease, those with diabetes, and those affected by Covid. This approach appears somewhat broad. Could you elucidate whether the aim is to establish a correlation among these conditions? If so, references to prior studies establishing such a relationship would significantly strengthen your argument. 

**AR**: This study's primary goal was to determine whether salivary CRP and IL-6 may be used as possible indicators of systemic inflammation in people with COVID-19-diagnosed periodontitis who have diabetes or not. As can be seen in Methods section, under the subtitle of

**Subjects, saliva sampling, and in vitro methods; “all COVID-19 patients participated in this study received a diagnosis of periodontitis. The patients were further split into two groups: those with (n = 10) and those without (n = 13) diabetes. As controls, six periodontitis non-diabetic individuals and no COVID-19 were also recruited”.**

 Additionally, in Methods section, under the subtitle of Data analysis, we explained; “The nonparametric Kruskal Wallis test was used to compare the diabetic group (DG), the non-diabetic group (NDG), and the control group. The Mann-Whitney test was used to compare the DG and NDG data”.

 Therefore, the diabetes group (DG), the non-diabetic group (NDG), and the control group were compared using the nonparametric Kruskal Wallis test, as we detailed in the Methods section.

**4. RC**: Control Group Discrepancy: There seems to be a contradiction regarding the control group's condition; the summary indicates they suffer from gum disease, whereas the methods section suggests they are free from any ailments. A consistent definition of the control group across all sections is crucial for the clarity and integrity of the study.

**AR**: I greatly appreciate this crucial correction. As a result, we changed the explanation of the control group in the Methods section to read as follows: As controls, six non-diabetic individuals with periodontitis but no COVID-19 were also included.

**5. RC**: Supporting Evidence for Conclusions: The current conclusions would benefit greatly from additional supporting evidence from relevant studies. Incorporating references and discussions around existing research would provide a more robust context for your findings.

**AR**: Thank you for your suggestions. Therefore, in the revised version we changed the sentence as following:

 According to the current study, salivary levels of CRP and IL-6, which have a low correlation, seems to be a valuable oral fluid biomarker that could be useful in differentiating between patients with COVID-19 who have diabetes and those who do not. Thus, corroborate a prior study that found a connection between periodontal diseases and the expression of proinflammatory cytokines (IL-6 and CRP) associated with hyperglycemia. ^45^ Furthermore, the results of saliva analytes (IL-6 and CRP) align with the presence of oral dysbiosis, which is indicated by the presence of periodontitis. ^4^ This implies that oral dysbiosis in diabetics may raise the risk of diabetes-associated inflammation in situations of mild SARS-CoV-2 infection, This suggests that in cases of mild SARS-CoV-2 infection, oral dysbiosis in individuals with diabetes might increase the probability of diabetes-associated inflammation, which is characterized by increased levels of salivary CRP and IL-6.

**6. RC**: Exploration of Limitations: I noticed an absence of a detailed discussion on the limitations of your study. Understanding and acknowledging these limitations is vital for a comprehensive view of the research's scope and impact.

**AR**: We agree to reviewer’s comments. Therefore, the related paragraph has been changed accordingly, as following: 

 There are certain restrictions on this observational study. Firstly, the subjects who were included were limited, therefore these results could not be indicative of the total population impacted by COVID-19. More research with a larger sample size is needed. Secondly, the study design did not account for the alterations in salivary proteins during the saliva collection process. Future research is required to evaluate dynamic salivary changes because the study's findings do not conclusively suggest a correlation between the level of inflammatory determinants in saliva and the systemic inflammatory mechanism of COVID-19. Finally, we cannot completely rule out the possibility that salivary flow rate influences the levels of IL-6 and CRP in saliva. Consequently, it is advised to validate the findings of this study and incorporate this confounding variable as a patient-specific component in subsequent research.

**7. RC**: Your attention to these matters would greatly enhance the clarity and depth of your study. I look forward to your revisions.

**AR**: Thank you.

# Reviewer report: Reviewer response for version 2

**DOI:** 10.5256/f1000research.157516.r218232

**Published:** 2023-11-02

**Authors:** Joel Schwartz

**Affiliations:** 1 University of Illinois Chicago, Chicago, USA

I stated previously and still think this study although only involving a small cohort does provide important data which needs additional study. The authors did attempt to incorporate my conceptual concerns in the discussion.

 Please index this study without additional edits.

Is the work clearly and accurately presented and does it cite the current literature?

Yes

If applicable, is the statistical analysis and its interpretation appropriate?

Yes

Are all the source data underlying the results available to ensure full reproducibility?

No

Is the study design appropriate and is the work technically sound?

Yes

Are the conclusions drawn adequately supported by the results?

Yes

Are sufficient details of methods and analysis provided to allow replication by others?

No

Reviewer Expertise:

Oral biology, molecular biology, biochemistry, pathology, mucosal immunology

I confirm that I have read this submission and believe that I have an appropriate level of expertise to confirm that it is of an acceptable scientific standard.

# Reviewer report: Reviewer response for version 1

**DOI:** 10.5256/f1000research.143798.r208201

**Published:** 2023-09-22

**Authors:** Joel Schwartz

**Affiliations:** 1 University of Illinois Chicago, Chicago, USA

Rationale for the study is pro-inflammatory markers such as IL-6 and CRP derived from the saliva are identifiable and associated with the presence of COVID-19 and/or possible periodontal disease.. Results show correlations to be present in the data rejecting the null hypothesis with a high level of probability. 

 However, the problem with these results are these.

 (relatively low number) 23 Samples (10 with diabetes mellitus (DM) and 13 with no (DM). We need information about the phase of disease pathogenesis and determine possible inflammatory behavior. regarding DM (e.g., Type 1 /Type 2, intermediate, active, control, uncontrolled, medication used? etc.) Periodontal disease (e.g., mild-moderate, sever, generalized, localized? How was PDS assessed: periodontal bone probing, indexing (plaque, gingival, bleeding, CAL/ALOSS), radiology? all or PSR shortcut?) 

 Saliva is a great source of inflammatory makers. Some of these are transudate or exudate products from the serum. Therefore, coincident levels between the saliva and serum can vary. The should clearly state in the discussion is SARS-CoV-2 is an oral virus. Its tropism is targeting oral epithelial cells populations based on the expression pattern of AVCE/TMPRSS2 which include keratinocytes, salivary ductal cells of various types which includes myoepithelial cells, tuft cells, gustatory and supporting cells of the tongue. Using saliva samples without gingival crevicular fluid as alluded to in the discussion increases difficulty to assess contribution by gingival tissues compared to other tissues. In addition, there are citations available that indicate there is a hierarchy of ACE2/TMPRSS2 expression with the highest levels in tongue and salivary gland coinciding with clinical signs such as loss of taste and dry mouth. These types of signs and symptoms need to be described to provide some degree of characterization of inflammation related to tissue site..

 Additionally, there is lacking a immunologic description of the functional activity of IL-6. This cytokine which is also described as a adipokine is suppressible by adiponectin derived from the saliva.  IL-6 has a variety of function requiring a mention in the discussion. For example, regulation of loss of tolerance and B cell differentiation while also effecting innate responses particularly in the periodontal tissues. Above I mentioned a possible association with adiponectin which acts as a protector against DM while increasing glucose uptake. There is another association overlooked in the discussion periodontal pathogens release metallo-peptidases and one of these mimics dipeptidyl-peptidase IV  (DDPIV)  which is diabetogenic. These types of relationship validate previous reports that periodontal disease is linked to DM. 

 I suggest providing additional information in methods section about the health conditions of the subjects.

 Try to provide additional markers from the serum of saliva identifying SARS-CoV_2 presence in the oral cavity.

 Update and edit the discussion to comply with knowledge of SARS-CoV_2 and COVID-19 presentation effecting oral tissues.

Is the work clearly and accurately presented and does it cite the current literature?

Yes

If applicable, is the statistical analysis and its interpretation appropriate?

Yes

Are all the source data underlying the results available to ensure full reproducibility?

No

Is the study design appropriate and is the work technically sound?

Yes

Are the conclusions drawn adequately supported by the results?

Yes

Are sufficient details of methods and analysis provided to allow replication by others?

No

Reviewer Expertise:

Oral biology, molecular biology, biochemistry, pathology, mucosal immunology

I confirm that I have read this submission and believe that I have an appropriate level of expertise to state that I do not consider it to be of an acceptable scientific standard, for reasons outlined above.

# Response

**Published:** 2023-10-13

**Authors:** Endang Bachtiar

Dear Reviewer,

 Thank you for giving us the opportunity to submit a revised version of the manuscript “The utility of salivary CRP and IL-6 as a non-invasive measurement evaluated in patients with COVID-19 with and without diabetes”. We appreciate the time and effort that the Reviewer dedicated to providing feedback on our manuscript and are grateful for the insightful comments on and valuable improvements to our paper. We have incorporated most of the suggestions made by the reviewer Those changes are highlighted in green within the manuscript.

 Please see below, for a point by point response to the reviewer’s comments and concerns. All page numbers refer to the revised manuscript with tracked changes.

 Reviewer’s Comments ( **RC**)

  

1. Rationale for the study is pro-inflammatory markers such as IL-6 and CRP derived from the saliva are identifiable and associated with the presence of COVID-19 and/or possible periodontal disease.. Results show correlations to be present in the data rejecting the null hypothesis with a high level of probability.

However, the problem with these results are these.

 (relatively low number) 23 Samples (10 with diabetes mellitus (DM) and 13 without no (DM).

 Author’s Response ( **AR**): Yes, we agree. As we mentioned in the limitation of our study; **page 9 line 33**. “This observational study has some limitations. First, the included participants were restricted. Additionally, as mentioned in Methods (under the subtitle: Subjects, saliva sampling, and in vitro methods). ……….we performed the study in accordance with the guidelines provided by the ethics committee of RSUI, including “how consent was obtained from participants” ( **Page 4, line 10**). Thus, only participants gave her consent was included.

1. **RC**: We need information about the phase of disease pathogenesis and determine possible inflammatory behaviour. regarding DM (e.g., Type 1 /Type 2, intermediate, active, control, uncontrolled, medication used? etc.) Periodontal disease (e.g., mild-moderate, sever, generalized, localized? How was PDS assessed: periodontal bone probing, indexing (plaque, gingival, bleeding, CAL/ALOSS), radiology? all or PSR shortcut?)

**AR**: Thank you for pointing this out. As can be seen in Methods section, all information about systemic condition of the patient, we obtained from the medical records.   In the revised version, we have added some important information regarding “clinical status”, such as age, sex, and chronic medical history of comorbidities, that was obtained from the medical reports (restricted) of mildly symptomatic patients with COVID-19 (not shown).

 Only subjects who had no respiratory symptom for more than 2 weeks were included in this study, However, our focus was on patients with COVID-19, with and without diabetes. Thus, only diabetic status (type 2 diabetes), as reported in the medical records, was included as a comorbidity variable in the data analysis, while all COVID-19 patients were diagnosed with periodontitis (moderate to severe) according to the criteria described by the American Academic of Periodontology Classification of Periodontal Disease ^6^. In this study, it would not possible to take dental radiology. Therefore, in revised manuscript, we added “without dental radiographic assessment following “patient diagnosed with periodontitis…….,” mentioned above ( **Page 4, Line 16-18**).

1. **RC**: Saliva is a great source of inflammatory makers. Some of these are transudate or exudate products from the serum. Therefore, coincident levels between the saliva and serum can vary.

The should clearly state in the discussion is SARS-CoV-2 is an oral virus. Its tropism is targeting oral epithelial cells populations based on the expression pattern of AVCE/TMPRSS2 which include keratinocytes, salivary ductal cells of various types which includes myoepithelial cells, tuft cells, gustatory and supporting cells of the tongue.

 Using saliva samples without gingival crevicular fluid as alluded to in the discussion increases difficulty to assess contribution by gingival tissues compared to other tissues. In addition, there are citations available that indicate there is a hierarchy of ACE2/TMPRSS2 expression with the highest levels in tongue and salivary gland coinciding with clinical signs such as loss of taste and dry mouth. These types of signs and symptoms need to be described to provide some degree of characterization of inflammation related to tissue site..

 Additionally, there is lacking a immunologic description of the functional activity of IL-6. This cytokine which is also described as a adipokine is suppressible by adiponectin derived from the saliva.  IL-6 has a variety of function requiring a mention in the discussion.

 For example, regulation of loss of tolerance and B cell differentiation while also effecting innate responses particularly in the periodontal tissues. Above I mentioned a possible association with adiponectin which acts as a protector against DM while increasing glucose uptake.

 There is another association overlooked in the discussion periodontal pathogens release metallo-peptidases and one of these mimics dipeptidyl-peptidase IV  (DDPIV)  which is diabetogenic. These types of relationship validate previous reports that periodontal disease is linked to DM.

**AR**: We agree and thank the reviewer to underline this important issue. Accordingly, in the revised manuscript, we have added explanations ( **Page 9, Line 9-34) **regarding the tropism of SARS-CoV-2 as an oral virus, the hierarchy of ACE2/TMPRSS2 expression in oral tissue, as well as the oral sign and symptoms related to COVID-19, the immunologic description of IL-6 function as mentioned by the Reviewer, as follows; 

 Current literatures show that IL-6 is a pleiotropic cytokine with complex role in inflammation and metabolic disease. Its biological activities include B-lymphocyte differentiation, T-lymphocyte proliferation ^13^,  pro and anti-inflammatory activities ^14^, the development of the nervous and hematopoietic system, and the regulation of metabolism ^15^. As a proinflammatory cytokine, it induces insulin resistance and periodontal disease in the process of bone resorption ^16^. Taken together, Il-6, which is considered an adipokine has an important role in the pathogenesis of localized oral inflammation (periodontitis), and may play a pivotal role in metabolic disease ^17^, such as diabetes.

   

 The current study is an extension of our primary study involving the same patients with COVID-19 to evaluate the inflammatory conditions in periodontal microenvironment ^18^. We found that the transcription levels of both IL-6 and complement C3 (the central component of innate immune system) in gingival crevicular fluid (GCF) were markedly higher in COVID-19 patients with diabetes (DG) compared to the non-diabetes patients (NDG). We also noticed that in periodontal niche, the mRNA upregulation of host receptor for SARS-CoV-2 (angiotensin-converting enzyme 2/ ACE2) ^19^was positively associated with the transcription levels of either inflammation marker tested (IL-6 or C3).   Thus, our previous results along with other work by other investigators ^20, 21^ suggest  that the virus receptor (ACE2) can be detected in oral tissue.  Additionally, certain molecules (furin and TMPRSS2) that are involved in promoting the SARS-CoV-2 entry and infection can be detected in oral cavity ^22^. This means that the essential molecules for SARCOV-2 infection are abundant in the oral cavity, and the infected virus may lead to localized inflammation and loss of taste (dysgeusia) as well as dry mouth, which are the most frequently reported symptom in COVID-19 patient ^23-25^. The literature search also indicated that the relationship between COVID-19 and diabetes mellitus is complicated and bidirectional ^26^. In addition to diabetes, all patients with COVID-19 included in the current study also had periodontitis, a common comorbidity observed in patients with COVID-19 ^27^.  

 For the involvement of peptidase produced by periodontopathic pathogen, and mimics DDP4/DDP1V molecule of eukaryote cells, we have added explanations that can be found in the Discussion section ( **Page 8, Line 8-13**), as follows;

 However, unlike salivary CRP, IL-6 levels in saliva have been reported to not correlate with those in plasma or serum ^30,31^. ^^In this regard, the elevated levels observed in this study may indicate inflammation induced by periodontitis-associated bacteria, particularly *Porphyromonas gingivalis*, as its components (dipeptidyl peptidase-4 /DPP4), that mimic human dpp4, involved in postprandial glycemic control in individuals with type 2 diabetes ^32, 33^, that according to the medical record, were the participants included in the current study.

1. **RC**: I suggest providing additional information in methods section about the health conditions of the subjects. Try to provide additional markers from the serum of saliva identifying SARS-CoV_2 presence in the oral cavity. Update and edit the discussion to comply with knowledge of SARS-CoV_2 and COVID-19 presentation effecting oral tissues.

**AR**: As mentioned in the above point 2, in the revised version of this manuscript, the additional information about participant’s health condition have been added. As suggested by the reviewer, we also added the additional marker for the serum of saliva (complement C3) by referring to our previous study ^18^. It can be seen in the above author response (point 3). Moreover, we have added the suggested content to the Discussion section, as explained in the above point 3.
